# Supplementary material for: Direct Learning Hidden Excited State Interaction Patterns from ab initio Dynamics and Its Implication as Alternative Molecular Mechanism Models
Source: Sci Rep. 2017 Aug 18;7:8737. doi: 10.1038/s41598-017-09347-2 (PMC5562909; doi:10.1038/s41598-017-09347-2)
Supplement: Supplementary file 1 — Supplementary Information [file 41598_2017_9347_MOESM1_ESM.pdf]

Supporting Information

Direct Learning Hidden Excited State Interaction Patterns from *ab initio* Dynamics and Its Implication as Alternative Molecular Mechanism Models

Fang Liu[1], Likai Du[1]*, Dongju Zhang[2], Jun Gao[1]*

[1]Hubei Key Laboratory of Agricultural Bioinformatics, College of Informatics, Huazhong Agricultural University, Wuhan, 430070, P. R. China

[2]Institute of Theoretical Chemistry, Shandong University, Jinan, 250100, P. R. China

*Correspondence to dulikai@mail.hzau.edu.cn; gaojun@sdu.edu.cn

Details of the Excited State Dynamics Simulations

The molecular conformation data sets were mainly collected from our previous excited state AIMD simulations of sinapic acids (SA).[1] Here, we only give a brief summary. The ultrafast excited-state AIMD was performed by on-the-fly surface hopping approach as implemented in JADE package.[2-3] The nuclear and electronic degrees of freedom are treated by classical and quantum dynamics, respectively. This approach is possible to treat relatively large and realistic molecular systems with full degree of freedom (DoF). The initial geometries and velocities of the excited state dynamics simulations were generated from the Wigner distribution function of the first vibrational level of the ground electronic state.[4-5] Starting from the initial sampling geometries, the molecule is electronically excited to the S_1 state for 100 trajectories. Each trajectory was calculated for 1000 fs. The time step for integration of classical equations was 0.5 fs and of quantum equations, 0.005 fs. The decoherence correction proposed by Granucci et. al. was taken and the parameter is set to α=0.1 Hartree[6]. For comparison, we also extended the dynamic simulation up to 10,000 fs for a few dynamics trajectories. Non-adiabatic transitions between excited states were taken into account via Tully's fewest switches approach[7]. The excited states molecular dynamics of sinapic acid (SA) was calculated using at B3LYP/6-31G(d,p) level, and the D3 dispersion correction is applied.

Reference

1. Liu, F.; Du, L.; Lan, Z.; Gao, J., Hydrogen bond dynamics governs the effective photoprotection mechanism of plant phenolic sunscreens. *Photochem. Photobiol. Sci.* **2017,** *16* (2), 211-219.

2. Du, L.; Lan, Z., An on-the-fly surface-hopping program jade for nonadiabatic molecular dynamics of polyatomic systems: implementation and applications. *J. Chem. Theory Comput.* **2015,** *11* (4), 1360-1374.

3. Du, L.; Lan, Z., Correction to An On-the-Fly Surface-Hopping Program JADE for Nonadiabatic Molecular Dynamics of Polyatomic Systems: Implementation and Applications. *J. Chem. Theory Comput.* **2015,** *11* (9), 4522-4523.

4. Barbatti, M.; Aquino, A. J.; Lischka, H., The UV absorption of nucleobases: semi-classical ab initio spectra simulations. *Phys. Chem. Chem. Phys.* **2010,** *12* (19), 4959-4967.

5. Mitrić, R.; Hartmann, M.; Stanca, B.; Bonačić-Koutecký, V.; Fantucci, P., Ab Initio Adiabatic Dynamics Combined with Wigner Distribution Approach to Femtosecond Pump−Probe Negative Ion to Neutral to Positive Ion (NeNePo) Spectroscopy of Ag2Au, Ag4, and Au4 Clusters. *J. Phys. Chem. A* **2001,** *105* (39), 8892-8905.

6. Granucci, G.; Persico, M., Critical appraisal of the fewest switches algorithm for surface hopping. *J. Chem. Phys.* **2007,** *126* (13), 134114.

7. Tully, J. C., Molecular dynamics with electronic transitions. *J. Chem. Phys.* **1990,** *93* (2), 1061-1071.

Details of K-means Clustering Algorithm

The K-means algorithm works as follows:

1) Every point is initially infinitely far from any cluster center;

2) choose an arbitrary point as the first cluster center;

3) compute the distance between every point and the new cluster center;

4) assign points to this new cluster center if they are closer to it than the cluster center they are currently assigned to;

5) Declare the point that is furthest from every cluster center to be the next new cluster center;

6) And repeat steps 2–5 until the desired number of clusters have been generated. The algorithm has complexity O (kN) where k is the number of clusters to be generated and N is the number of data points to be clustered.

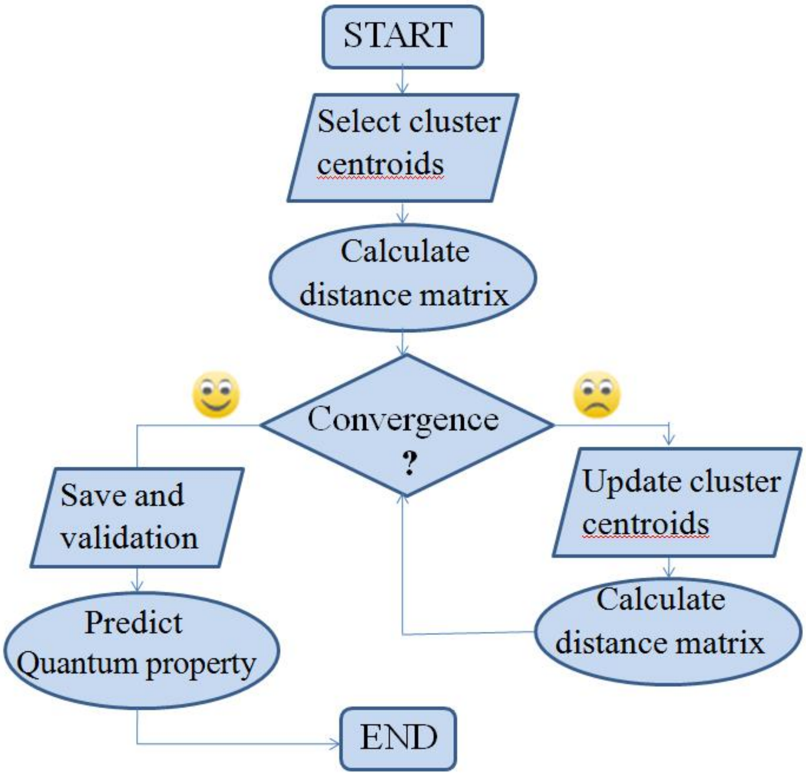

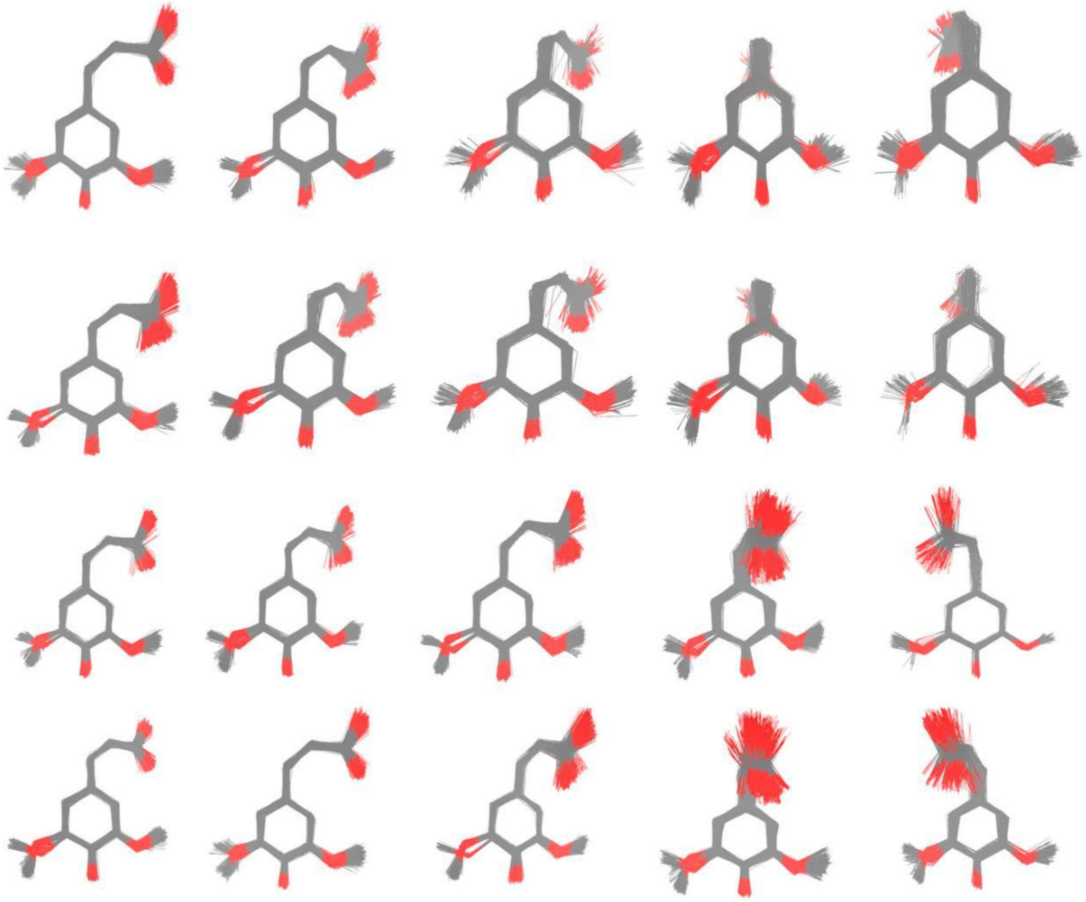

Figure S1. Possible meta-stable patterns derived from K-means clustering results (k=20). The oxygen and carbon atoms are shown in red and gray, and the position of the hydrogen atom is not shown for clarity.

Table S1. The number of sampled snapshots (%) in each meta-stable pattern, after the K-means clustering algorithm. We only sample the local minimum along each trajectory (13226 structures).

cluster	%	cluster	%
1	13.3	1'	11.0
2	8.5	2'	9.3
3	8.1	3'	9.0
4	10.0	4'	8.2
5	7.3	5'	7.3
6	4.2	6'	3.8

Table S2. The mean, minimum and maximum value of molecular structure RMSD for each meta-stable pattern. Only the non-hydrogen atom coordinates are considered in the RMSD calculations.

cluster	Mean value (Å)	Minimum value (Å)	Maximum value (Å)
1	0.28	0.13	0.57
2	0.32	0.14	0.61
3	0.31	0.13	0.65
4	0.30	0.11	0.76
5	0.38	0.16	0.96
6	0.41	0.18	0.82
1'	0.28	0.12	0.54
2'	0.29	0.12	0.63
3'	0.30	0.09	0.61
4'	0.30	0.10	0.70
5'	0.36	0.15	0.85
6'	0.41	0.21	0.81

Optimized coordinates of SA molecule on the ground state

C	2.05527800	0.73480200	-0.00014000
C	2.17308000	-0.66659400	-0.00015200
C	1.01864800	-1.44694000	-0.00003800
C	-0.26759000	-0.85977200	0.00008400
C	-0.37704600	0.54418600	0.00008500
C	0.77458900	1.31678300	-0.00003600
H	1.10164400	-2.52698500	-0.00005400
H	-1.35897000	0.99166900	0.00018500
O	3.16983900	1.50432000	-0.00022800
H	2.86783700	2.42690000	-0.00017800
O	0.80899500	2.69007000	-0.00006200
O	3.45103500	-1.14241800	-0.00031100
C	-1.39764700	-1.77689000	0.00020600
C	-2.75211500	-1.65874200	0.00022200
H	-1.07251400	-2.81752800	0.00030900
C	-3.61624200	-0.47433700	0.00010100
H	-3.31511600	-2.58643900	0.00028000
O	-4.92593800	-0.85587000	-0.00044900
H	-5.43880700	-0.03136000	-0.00070300
O	-3.31973600	0.71254100	-0.00005400
C	-0.43554300	3.38356800	0.00034000
H	-0.18848700	4.44600700	0.00038900
H	-1.02624800	3.14160800	-0.89106500
H	-1.02578000	3.14141200	0.89200400
C	3.63547500	-2.54830400	0.00034600
H	3.20162300	-3.01649700	-0.89371200
H	4.71454000	-2.70880900	0.00053600
H	3.20140300	-3.01567900	0.89472400

Optimized coordinates of SA molecule on the first excited state (S_1)

C	-1.81135800	0.13129900	-0.04833300
C	-1.35379600	-1.20381200	0.23134200
C	-0.07881100	-1.36728400	0.77635800
C	0.74371300	-0.28439400	1.07256800
C	0.24767800	1.04770800	0.83794500
C	-0.97685100	1.24351400	0.26693700
H	0.27600300	-2.37645200	0.94032600
H	0.90892300	1.87035200	1.06755200
O	-3.00083200	0.36483900	-0.61303900
H	-3.06107900	1.33265500	-0.73800000
O	-1.56069100	2.42916700	-0.08176700
O	-2.05470200	-2.32023000	0.00414500
C	2.10952600	-0.46920800	1.63295700
C	3.18453100	-0.66195100	0.74883400
H	2.16236800	-0.81426400	2.65857700
C	3.09810800	-0.33543700	-0.62023900
H	4.14148200	-0.99712100	1.13283000
O	4.26669200	-0.53935200	-1.34770900
H	4.02330400	-0.25861300	-2.24174500
O	2.08715800	0.12142100	-1.22025400
C	-0.73925900	3.60187100	-0.04945200
H	0.16083400	3.45472600	-0.65491300
H	-1.34708600	4.40557600	-0.46447700
H	-0.45749300	3.84715400	0.98013300
C	-3.36469900	-2.33832000	-0.58590400
H	-3.34673300	-1.90909100	-1.58990200
H	-3.63262800	-3.39313700	-0.63123300
H	-4.08158800	-1.79245600	0.03177700
